# Does the EQ-5D-5L benefit from extension with a cognitive domain: Testing a multi-criteria psychometric strategy in trauma patients

**DOI:** 10.1007/s11136-020-02496-4

**Published:** 2020-04-10

**Authors:** A. J. L. M. Geraerds, Gouke J. Bonsel, Suzanne Polinder, M. J. M. Panneman, M. F. Janssen, Juanita A. Haagsma

**Affiliations:** 1 Department of Public Health, Erasmus MC, University Medical Center Rotterdam, P.O. Box 2040, 3000 CA Rotterdam, The Netherlands; 2 Consumer and Safety Institute, Amsterdam, the Netherlands; 3 Section Medical Psychology and Psychotherapy, Department of Psychiatry, Erasmus MC, Rotterdam, The Netherlands

**Keywords:** HRQL, EQ-5D-5L, Cognition, Bolt-on, Responsiveness

## Abstract

**Purpose:**

This study investigated the psychometric yield of extension of the EQ-5D-5L with a cognitive domain (EQ-5D+C) in a mixed cohort of trauma patients with repeated data.

**Methods:**

A stratified sample of patients that presented at the emergency department filled out a follow-up survey 6 and 12 months after trauma. The surveys included the EQ-5D-5L+C, EQ-VAS, and the impact of events scale-revised (IES-R), a validated post-traumatic stress disorder (PTSD) self-assessment scale. Generally, results of the EQ-5D and EQ-5D+C were compared. Psychometrics included the following: distributional features (ceiling/floor effects), discriminatory performance, convergent validity with the EQ-VAS as reference, and responsiveness to change. Psychometric properties were compared between predefined subgroups based on conditions with cognitive impact (Traumatic Brain Injury (TBI)/PTSD).

**Results:**

In total, 1799 trauma patients responded 6 and 12 months after trauma, including 107 respondents with PTSD, and 273 with TBI. Six months post-trauma, ceiling of the EQ-5D (26.3%) was reduced with 2.2% with the additional cognitive domain. Using EQ-VAS as reference, convergent validity increased slightly with the addition of the cognitive domain: correlation increasing from 0.651 to 0.664. Cognitive level was found to slightly improve over time in TBI (delta: 0.04) and PTSD patients (delta: 0.05), while (almost) no change was found in patients without TBI and PTSD.

**Conclusion:**

Adding a cognitive domain to the EQ-5D-5L slightly improved measurement properties and better captured change in health status for trauma patients with TBI and PTSD. Inclusion of the cognitive domain in the EQ-5D-5L when measuring in populations with cognitive problems should be considered.

## Background

Measuring health-related quality of life (HRQL) is an important aspect of research in trauma patients [1]. HRQL can be measured with either a generic or a disease-specific measurement instrument [2]. One of the most widely used preference-based generic health status instruments is the EQ-5D [3]. The EQ-5D is a measurement instrument based on self-assessment that consists of five domains: mobility, self-care, usual activities, pain/discomfort and anxiety/depression [4]. Each domain contains one item that informs on the degree of problems. The major advantage of the EQ-5D compared to other generic instruments is its conciseness, and therefore low burden to complete [5, 6]. Ordinal response scales to the EQ-5D have either 3 levels (EQ-5D-3L) or 5 levels (EQ-5D-5L) [3].

The EQ-5D-3L is known to show a ceiling distribution, which implies that the ability to measure small changes in the upper part of the health scale is limited [7]. Stated otherwise: the instrument does not artificially increase distances in the upper part of the scale for the sake of discrimination. The three-level version and five-level version have been compared in previous studies to determine whether a 5-level response scale adds value to the measurement instrument. Janssen et al. [8], for example, reported that most measurement properties improved with the EQ-5D-5L. Only few studies compared head-to-head the responsiveness (sensitivity to change) of the three- versus five-level version [9–12]. The results of these studies were contradictory, as responsiveness did not improve in the five-level version in two of the studies [9; 10], while it did improve in the study by Buchholz et al. and Janssen et al. [11, 12].

Apart from extending the EQ-5D response categories from three to five to improve sensitivity, previous studies have also suggested that additional domains to the EQ-5D-3L, so-called ‘bolt-ons’, may improve the measurement properties in both general and specific populations [13–16]. One of the proposed bolt-ons, which is often already added to the EQ-5D, is a cognitive domain (EQ-5D-3L+C). Various studies showed that measurement properties improved slightly if the cognitive domain is added to the EQ-5D-3L when comparing EQ-5D and EQ-5D+C cross-sectionally [17, 18]. One of these studies assessed the added value of the cognitive domain in a sample of trauma patients in the Brabant region of the Netherlands who were admitted to the hospital due to a traumatic brain injury (TBI) [17]. However, measurement properties and responsiveness of the EQ-5D-5L extended with cognition in a general trauma population, including patients with cognitive conditions (such as TBI, but also post-traumatic stress disorder (PTSD)), has not been studied using the current methodology developed for 3L/5L comparisons. Therefore, in this study, we investigated the added value of the cognitive domain using supplementary psychometric analysis and more recently collected repeated follow-up data from trauma patients.

The aim of this study was to investigate the convergent validity, descriptive dependency and responsiveness of the EQ-5D-5L and the EQ-5D-5L+C in a comprehensive sample of trauma patients.

## Methods

### Research population

This study was conducted with data from the ‘Letsel informatie systeem’ (LIS) (Dutch injury patient surveillance system). LIS is an ongoing data collection in fourteen (out of approximately 90) hospitals in the Netherlands [19]. The fourteen hospitals are a representative sample of hospitals in the Netherlands and consist of academic and non-academic and rural and urban hospitals. The LIS hospitals register information on age, sex, circumstances of trauma, cause of trauma, in-hospital health care consumption, such as length of hospital stay and nature of trauma of each patient that visits the emergency department due to a trauma or poisoning [20]. After treatment of trauma, the patient was admitted to the hospital or discharged to the home environment or institution. The data used in this study were limited to the data collected in 2017. A stratified sample of patients received questionnaires 6 months and 12 months after trauma, with exclusion criterion for participation < 15 years old. The first questionnaire contained an informed consent form. Approval by the Medical Ethics Review Committee (METC) was not required according to the METC of the Academic Medical Center of the University of Amsterdam. The questionnaires contained questions on education level, comorbidity and HRQL. HRQL was measured with the EQ-5D-5L with cognitive domain and with the EQ-VAS.

### HRQL data

The EQ-5D-5L is a measurement instrument that consists of five domains with each five response levels. The five domains are mobility, self-care, usual activities, pain/discomfort and anxiety/depression. Cognition was added as a sixth domain to the five existing domains. The response options of the 5-level version are no problems, slight problems, moderate problems, severe problems, and extreme problems/unable to. Responses to the five EQ-5D and six EQ-5D+C domains can be combined in so-called health profiles, which define the severity level, where 1 means no problems and 5 means extreme problems or unable to perform, e.g. ‘21421′ for EQ-5D and ‘132512′ for EQ-5D+C. An unweighted summary score (level sum score) can be calculated from the responses to the EQ-5D, ranging from 5 to 25 for the EQ-5D and 6 to 30 for the EQ-5D with cognition. To enable comparison between the two level sum scores, scores on the EQ-5D+C were recoded to the same scale as scores on the EQ-5D by multiplying the level sum score with 5/6. In addition to the six domains of the EQ-5D, the EQ-VAS is also part of the EQ-5D. The EQ-VAS requires respondents to rate their health on a scale from 0 to 100, where 0 represents the worst imaginable health state, and 100 represents best imaginable health. Furthermore, the impact of events scale-revised (IES-R), which informs on PTSD-related complaints, was included in the questionnaire. The IES-R is a self-report questionnaire that consists of 22 items, which measure intrusive re-experiences of the trauma, avoidance of trauma-related stimuli and hyper arousal symptoms [21]. By combining the 22 items the total IES-score, ranging from 0 through 88, can be calculated. The cut-off score was set at 33, as advised by Creamer, Bell and Failla [22], with scores below 33 representing no PTSD, and scores equal to or higher than 33 representing PTSD. Data were imputed for respondents with one or two missing items on the IES-R using simple imputation, based on responses to other items of the IES-R. In addition, the first questionnaire included 19 items regarding the presence of one or more chronic diseases prior to trauma to assess comorbidity. Comorbidity is defined as the self-reported presence of any co-existing medical diseases or disease processes additional to the trauma that the trauma patients sustained.

### Data analysis

Data analyses were performed in SPSS version 25. Respondents were included in the analyses if an answer was provided for the EQ-5D+C and the EQ-VAS both 6 months and 12 months after trauma. The presence of traumatic brain injury (TBI) was determined based on the trauma registration in the LIS, and comorbidity was determined based on information from the follow-up questionnaire at 6 months. Comorbidity was divided in categories: no comorbidity; one comorbidity; two or more comorbidities. Furthermore, presence of PTSD was determined based on responses to the IES-R, which was included in the questionnaire at 6 months. Frequencies of socio-demographic characteristics were compared between responders and non-responders using Chi-square tests and Mann–Whitney U tests. A distributional effect in terms of ceiling was determined by defining the proportion of perfect health profiles (11111 for EQ-5D and 111111 for EQ-5D+C) among all observed profiles. A higher proportion of perfect health profiles indicates more ceiling.

Informativity, expressed as classification power of EQ-5D and EQ-5D+C was determined with the Shannon Index (*H′*) and Shannon Evenness Index (*J′*) [23]. Information on the ability to measure diversity in a population can be derived from these two indices [24]. To calculate the Shannon Index, the formula: *H′* = − ∑^c^_*i*=1_ *p*_*i*_^2^log *p*_*i*_ was used, where p_i_ represents the proportion of people with one health profile, and C represents the total number of possible health profiles. A higher value of *H*′ indicates that more information is captured by the measurement instrument. For EQ-5D, the total number of possible health profiles was 3125 (5 × 5 × 5 × 5 × 5), whereas for EQ-5D+C there were 15,625 possible health profiles. Next, the Shannon Evenness Index was calculated, based on the Shannon Index: *J*′ = *H*′/*H*′_max_, with *H*′_max_ representing ^2^log*C* (total number of possible health profiles). A higher value on the Shannon Evenness Index represents the capture of more information by the extra domain, and therefore increases the distinction between patients [8]. As the assessment of *H*′ using a sample of the total population will lead to an underestimation according to Pielou [25], adjusted values were calculated for *H*′ and *J*′ to control for this. Adjustment magnitude was set at (*C* − 1)/2*N*. Classification efficiency was determined for subgroups of respondents with TBI, PTSD and neither TBI nor PTSD, both for 6-month and 12-month measurement.

Furthermore, convergent validity of the EQ-5D-5L and the EQ-5D-5L+C was determined by the strength of association between the EQ-5D-5L with and without cognition with the EQ-VAS. First, the level sum score was calculated for both EQ-5D and EQ-5D+C as the sum of all domains (e.g. health profile 33333 had a level sum score of 15). The level sum score ranges from 5 to 25 for EQ-5D and 6 to 30 for EQ-5D+C, with a higher score representing poorer health. Subsequently, after confirmation that the assumptions were met, Spearman’s rank correlation coefficients between EQ-5D (all versions) and EQ-VAS were determined for the 6-month and 12-month assessments.

Additionally, explanatory power of EQ-5D and EQ-5D+C were determined using multivariable linear regression analyses, as the assumptions of linear regression were met, with EQ-VAS as dependent variable. Independent variables consisted of dummy variables for the levels ‘slight problems, moderate problems, severe problems, and extreme problems/unable to’ for all EQ-5D domains including cognition for the EQ-5D+C, with ‘no problems’ as the reference category. All combinations of 5 out of the 6 EQ-5D+C domains were analysed separately for TBI/PTSD respondents and for respondents with neither TBI nor PTSD.

### Discriminatory power

Mean level sum score was determined for both EQ-5D and EQ-5D+C at 6-month measurement. Scores were compared between subgroups of TBI/PTSD versus no TBI/PTSD, and no comorbidity versus one comorbidity versus two or more comorbidities, respectively. The level sum scores of EQ-5D and EQ-5D+C were compared within each subgroup using paired t-tests. Furthermore, level sum scores were compared between subgroups using one-way ANOVA.

### Responsiveness

Mean difference between reported EQ-5D and EQ-5D+C responses collected at 6 months and 12 months post-trauma were analysed per domain to gain insight into the average change per domain over time. The mean difference was calculated per domain by subtracting the 6-month score from the 12-month score: $\frac{D_{12}-D_{6}}{N},$ where* D*_12_ represents the score on the domain at 12 months,* D*_6_ the score on the same domain at the 6-month assessment, and *N* the total number of respondents. This resulted in a score between − 4 and 4.

Furthermore, EQ-5D and EQ-5D+C health profiles were compared at 6 months and 12 months using the Paretian classification of Health Change [26]. Based on the difference between the two measurement moments, respondents were classified as follows: no change; no problems; improved health; worsened health; non-categorisable (mixed change). Difference in classification between EQ-5D and EQ-5D+C was compared for subgroups of the population, based on the presence of TBI and PTSD.

To quantify change over time per domain, the probability of superiority was calculated by dividing the number of respondents with positive change (in terms of health improvement over time) by the total sample size. Half of the respondents that were categorised as ‘no change’ were added to the number of respondents with positive change to account for ties. Both the Paretian classification of Health Change and the probability of superiority can be interpreted as measures of responsiveness to change.

### Hypotheses

The following hypotheses were formulated:

- The ceiling effect is smaller with the EQ-5D+C compared to the EQ-5D;
- The convergent validity of the EQ-5D+C with the EQ-VAS is comparable to the EQ-5D with the EQ-VAS;
- Level sum score of the EQ-5D+C is differing from the level sum score on the EQ-5D in respondents with TBI or PTSD;
- Change in cognition is expected to be present in a larger percentage of respondents with TBI or PTSD than in respondents with neither TBI nor PTSD;
- The number of respondents with improved health in the Paretian Classification of Health is expected to increase with the addition of a cognitive domain in the group of TBI respondents, whereas the number of respondents with mixed change is expected to increase in the PTSD group.

## Results

### Research population

In total, 3941 respondents that presented to one of the hospitals included in the LIS filled out at least one questionnaire. Out of these 3941 respondents, 1799 completed the EQ-5D+C and the EQ-VAS at both 6 and 12 months after trauma. The socio-demographics of responders and non-responders are reported in Table 1. Within the group of responders, 273 respondents (15.2%) were diagnosed with TBI, and 107 (5.9%) reported PTSD, of which 18 respondents reported both TBI and PTSD. Comparing responders to non-responders, there were significant differences in age, education level, number of chronic conditions, hospitalisation and presence of PTSD (*p* < 0.05).

**Table 1:** Demographics of responders and non-responders to the EQ-5D+C and EQ-VAS at 6 and 12 months after trauma

Demographics of research population	Responders	Non-responders	*p* value
*n*	1799	2142	
Mean age (SD)	56.8 (23.2)	48.7 (29.3)	< 0.001*
Females	981 (54.5%)	1161 (54.2%)	0.837
*Education level n (*%*)*			< 0.001*
Low education	713 (39.6%)	825 (38.5%)	
Middle education	380 (21.1%)	347 (16.2%)	
High education	411 (22.8%)	338 (15.8%)	
* Missing*	295 (16.4%)	632 (29.5%)	
*Chronic conditions n (*%*)*			0.001*
0	1031 (57.3%)	1316 (61.4%)	
1	461 (25.6%)	460 (21.5%)	
≥ 2	269 (15.0%)	274 (12.8%)	
* Missing*	38 (2.1%)	92 (4.3%)	
Hospitalisation *n* (%)	923 (51.3%)	958 (44.7%)	< 0.001*
PTSD *n* (%)	107 (5.9%)	110 (5.1%)	< 0.001*
*Missing*	212 (11.8%)	527 (24.6%)	
TBI *n* (%)	273 (15.2%)	305 (14.2%)	0.408
*EQ-5D-5L (*+ *C) scores*^*a*^			
Mobility (*n* (%) with problems)	789 (43.9%)		
Self-care (*n* (%) with problems)	374 (20.8%)		
Usual activities (*n* (%) with problems)	936 (52.0%)		
Pain/Discomfort (*n* (%) with problems)	1176 (65.4%)		
Anxiety/Depression (*n* (%) with problems)	460 (25.6%)		
Cognition (*n* (%) with problems)	472 (26.2%)		
EQ-VAS (SD)	74.8 (18.47)		
Level sum score EQ-5D (SD)^b^	8.4 (3.56)		
Level sum score EQ-5D+C (SD)^b^	9.8 (3.94)		
Health profile 11111 (*n* (%))	473 (26.3%)		
Health profile 111111 (*n* (%))	434 (24.1%)		

*SD* standard deviation, *PTSD* post-traumatic stress disorder, *TBI* traumatic brain injury

^*^Significant at a 5% level (*p* < 0.05)

^a^EQ-5D (+ C) measured at 6 months post-trauma

^b^Level sum score was used, not utility

### Distributional effects

**Fig. 1:**
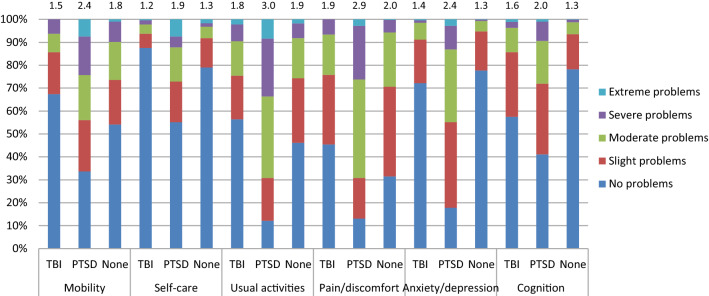
Percentage of respondents per level per domain for subgroups of TBI, PTSD and neither, including mean level scores per subgroup per domain. NOTE: Responders can be represented in two categories: TBI and PTSD

Figure 1 provides an overview of the percentage of respondents that reported problems per domain at 6-month measurement, for subgroups of patients with TBI, patients with PTSD and patients with neither TBI nor PTSD. Respondents with PTSD had the highest percentage of reported problems and mean level score on all domains.

For the EQ-5D, 473 out of 1799 respondents (26.3%) reported full health (no problems on any domain) 6 months after trauma, versus 434 out of 1799 (24.1%) on the EQ-5D+C. Looking at the subgroups, full health was found on the EQ-5D for 99 out of 273 (36.3%) TBI patients, versus 79 out of 273 (28.9%) for EQ-5D+C. In the group of patients with PTSD one out of 107 (0.9%) patients reported full health for both EQ-5D and EQ-5D+C. Considering patients with neither TBI nor PTSD, 295 out of 1254 (23.5%) patients for EQ-5D versus 278 out of 1254 (22.2%) for EQ-5D+C reported full health.

### Classification efficiency: Shannon indices

In total, 308 out of 3125 (9.9%) health profiles were reported for EQ-5D, and 460 out of 15,625 (2.9%) for EQ-5D+C. Comparing the diversity of the different subgroups (respondents with TBI, PTSD, and respondents with neither TBI nor PTSD), taking sample size into account, we found that respondents with PTSD showed the highest Shannon indices (*H*′ = 6.32 and *J*′ = 0.54 for EQ-5D and *H*′ = 6.60 and *J*′ = 0.47 for EQ-5D+C), indicating that the group of respondents with PTSD was more heterogeneous in terms of health profiles (Table 2). For each subgroup, the Shannon Evenness Index was higher for the EQ-5D than for the EQ-5D+C, indicating that more true information is captured. However, after adjusting for underestimation bias, the Shannon Evenness Index was higher for the EQ-5D+C than for the EQ-5D. This indicates that more information is captured in the EQ-5D+C.

**Table 2:** Shannon Index (*H*′) and Shannon Evenness Index (*J*′) of EQ-5D and EQ-5D+C 6 months after trauma

	Health profile	*H*′	Adjusted^a^ *H*′	*J*′	Adjusted^a^ *J*′
All	EQ-5D	5.74	6.61	0.49	0.57
	EQ-5D+C	6.37	10.71	0.46	0.77
TBI	EQ-5D	4.60	10.32	0.40	0.45
	EQ-5D+C	5.27	33.89	0.38	2.43
PTSD	EQ-5D	6.32	20.92	0.54	1.80
	EQ-5D+C	6.60	79.61	0.47	5.72
None^b^	EQ-5D	5.62	6.86	0.48	0.59
	EQ-5D+C	6.16	12.39	0.44	0.89

^a^Adjusted for underestimation bias

^b^None = No TBI and no PTSD

### Convergent validity

Convergent validity was higher for EQ-5D+C with EQ-VAS than for EQ-5D with EQ-VAS for all comparison groups (see Table 3). A negative relation was found as the level sum score decreases with better health while the EQ-VAS increases with better health. The convergent validity for the group of respondents with PTSD is much lower compared to the other subgroups.

**Table 3:** Spearman’s Rank Correlation for level sum score EQ-5D with EQ-VAS and level sum score EQ-5D+C with EQ-VAS at 6-month measurement for subgroups of TBI, PTSD and neither

Group	Variables	Spearman’s Rank Correlation [CI]
All	EQ-5D+EQ-VAS	− 0.651 [− 0.679, − 0.620]
	EQ-5D+C+EQ-VAS	− 0.664 [− 0.693, − 0.634]
TBI	EQ-5D+EQ-VAS	− 0.628 [− 0.701, − 0.541]
	EQ-5D+C+EQ-VAS	− 0.640 [− 0.717, − 0.556]
PTSD	EQ-5D+EQ-VAS	− 0.471 [− 0.622, − 0.283]
	EQ-5D+C+EQ-VAS	− 0.491 [− 0.639, − 0.302]
None^a^	EQ-5D+EQ-VAS	− 0.616 [− 0.653, − 0.577]
	EQ-5D+C+EQ-VAS	− 0.630 [− 0.667, − 0.592]

^a^None = No TBI and no PTSD, CI 95% confidence interval

### Explanatory power

Generally, 41% of the variance of the EQ-VAS could be explained in TBI and PTSD patients by 5 domains of the EQ-5D+C (Table 4). For respondents with neither TBI nor PTSD 40% of the variance could be explained. The percentage of explained variance increased slightly with the addition of the cognitive domain for both groups. Explained variance was highest for the group with neither TBI nor PTSD when all six domains were included, whereas it was highest for the TBI/PTSD group with the domains mobility, self-care, usual activities, pain/discomfort and cognition.

**Table 4:** Explanatory power of multivariable models for EQ-VAS with any combination of five of the EQ-5D+C domains for TBI/PTSD and neither TBI nor PTSD respondents

Group	Variables	*R*^2^ adjusted	*F* value	*p* value
TBI/PTSD	MO, SC, UA, PD, AD	0.413	13.68	< 0.001*
	MO, SC, UA, PD, AD, CO	0.415	11.66	< 0.001*
	MO, UA, PD, AD, CO	0.416	13.85	< 0.001*
	MO, SC, PD, AD, CO	0.393	12.67	< 0.001*
	MO, SC, UA, AD, CO	0.402	13.15	< 0.001*
	MO, SC, UA, PD, CO	0.418	13.98	< 0.001*
	SC, UA, PD, AD, CO	0.411	13.61	< 0.001*
None^a^	MO, SC, UA, PD, AD	0.401	42.97	< 0.001*
	MO, SC, UA, PD, AD, CO	0.414	37.91	< 0.001*
	MO, UA, PD, AD, CO	0.404	43.47	< 0.001*
	MO, SC, PD, AD, CO	0.389	40.82	< 0.001*
	MO, SC, UA, AD, CO	0.403	43.27	< 0.001*
	MO, SC, UA, PD, CO	0.408	44.21	< 0.001*
	SC, UA, PD, AD, CO	0.403	43.21	< 0.001*

*MO* mobility, *SC* self-care, *UA* usual activities, *PD* pain/discomfort, *AD* anxiety/depression, *CO* cognition

^a^No TBI and no PTSD

^*^Significant at a 5% level (*p* < 0.05)

### Discriminatory power

Mean level sum scores of EQ-5D and EQ-5D+C (adjusted for extra domain by multiplying with 5/6) were found to differ significantly (*p* < 0.05) for all groups, except for TBI patients (*p* = 0.211) (Table 5). Comparing level sum scores of the EQ-5D between respondents with TBI, respondents with PTSD and respondents with neither TBI nor PTSD, it was also found that scores differ significantly. The same applies to a comparison between groups of patients with no, one, and two or more chronic conditions.

**Table 5:** Mean level sum score for EQ-5D and EQ-5D+C for two subgroup classifications at 6-month measurement

Group	Level sum score EQ-5D	Level sum score EQ-5D+C	*p* value
All	8.4	8.2	< 0.001*
TBI	7.8	7.8	0.211
PTSD	12.6	12.1	< 0.001*
None^a^	8.4	8.1	< 0.001*
*p* value	< 0.001*	< 0.001*	–
No chronic condition	7.3	7.2	< 0.001*
1 chronic condition	9.4	9.1	< 0.001*
≥ 2 chronic conditions	10.9	10.4	< 0.001*
*p* value	< 0.001*	< 0.001*	–

^a^None = No TBI and no PTSD

*Significant at a 5% level (*p* < 0.05)

### Responsiveness to change

Table 6 provides an overview of the percentage of respondents per subgroup (TBI, PTSD, neither TBI nor PTSD) per change category for each domain. For all domains, the majority of patients (> 50%) showed no change. One exception is the group of respondents with PTSD, which shows a percentage below 50% with no change on both usual activities (36.4%) and anxiety/depression (49.5%). Furthermore, respondents with PTSD had, compared to the other subgroups, most often a change of one level over time (either positive or negative).

**Table 6:**
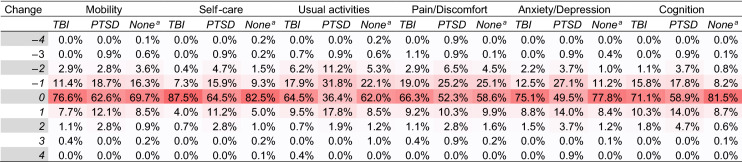
Frequency per change score over time (6 month–12 month) per domain per subgroup for the level sum score of the EQ-5D and EQ-5D+C

NOTE1: A darker shaded cell indicates a higher value. NOTE2: A negative value for change represents an improvement in health

^a^None = No TBI and no PTSD

According to the Paretian classification of Health changes (see “Methods” section), we observed that approximately a third of respondents improved over time (Table 7, row 'Improve'). The percentage of respondents that universally improved in all domains was slightly lower in the EQ-5D-5L+C, while the percentage of respondents with mixed results in domains was a little higher. This indicates that while improvement or stability was found for the domains of the EQ-5D-5L, there was deterioration in the cognitive domain for some respondents (resulting in the mixed, non-categorisable change group). Results in clinical subgroups based on TBI, PTSD and no PTSD or TBI were similar. Change in classification between EQ-5D and EQ-5D+C can be explained by a change in the cognitive domain in another direction than change on the other domains, which was clearly noted in the group of respondents with PTSD. Here, the mixed change response was 26.2% in EQ-5D vs. 40.2% in EQ-5D+C. Furthermore, for TBI patients there was a 6.9% decrease in patients with no problems and a 1.8% increase in the ‘improve’ category when the cognitive domain was included.

**Table 7:** Health (EQ-5D, EQ-5D+C) profile change over time according to Paretian classification for profile changes, for all trauma patients and by subgroup

Paretian classification of health change	All		TBI		PTSD		None^a^	
	EQ-5D	EQ-5D+C	EQ-5D	EQ-5D+C	EQ-5D	EQ-5D+C	EQ-5D	EQ-5D+C
No problems	22.1%	20.1%	32.2%	25.3%	–	–	19.3%	18.0%
No change (including no problems)	30.2%	27.0%	38.1%	31.2%	5.6%	3.7%	28.5%	25.9%
Improve	39.5%	38.2%	32.6%	34.4%	46.7%	38.3%	42.7%	41.1%
Worsen	16.5%	16.6%	15.0%	15.4%	21.5%	17.8%	16.3%	16.7%
Non-categorisable (mixed change)	13.8%	18.2%	14.3%	19.0%	26.2%	40.2%	12.5%	16.3%
Total *n*	1799		273		107		1254	

^a^None = No TBI and no PTSD

Domain wise, the probability of a positive change over time showed most improvement in usual activities and pain/discomfort (58%) and least improvement in cognition (50%) (Table 8). Subgroup analysis showed similar results for usual activities and pain/discomfort; however, cognition was found to have a higher probability of superiority in respondents with TBI and respondents with PTSD than in respondents with neither TBI nor PTSD.

**Table 8:** Probability of superiority per domain of the EQ-5D+C for study population

Domain	PoS			
	All	TBI	PTSD	None^a^
Mobility	0.55	0.53	0.54	0.56
Self-care	0.52	0.51	0.54	0.53
Usual activities	0.58	0.57	0.62	0.59
Pain/discomfort	0.58	0.56	0.60	0.59
Anxiety/depression	0.51	0.52	0.57	0.51
Cognition	0.50	0.52	0.52	0.50

*PoS* probability of superiority

^a^None = No TBI and no PTSD

## Discussion

### Main findings

This study investigated the potential gain of adding a cognitive domain to the EQ-5D-5L and analysed the distributional effects, construct validity and responsiveness to change of the instrument in a group of trauma patients with measurement of HRQL at 6 and 12 months after trauma. The addition of a cognitive domain slightly decreased the percentage of respondents reporting full health, which was in line with our first hypothesis. It also increased the adjusted Shannon Index and adjusted Shannon Evenness Index at both 6-month and 12-month measurement. Convergent validity and explanatory power of the EQ-5D with the EQ-VAS increased slightly with the addition of cognition, as expected. Furthermore, the level sum score of the EQ-5D and EQ-5D+C for subgroups based on TBI or PTSD presence and on number of chronic conditions differed significantly, which was in line with the third hypothesis, except for TBI patients. Results of the responsiveness to change analyses showed that change over time in the cognitive domain was, especially in respondents with PTSD, often in reverse direction of change in other domains. This can be explained by findings of previous studies among trauma patients that showed that PTSD symptoms can exacerbate over time [27–29]. In TBI patients on the other hand, it was found that the percentage of respondents with improved health increased with the inclusion of the cognitive domain. This was in line with the fifth hypothesis, and can be explained by the expected improvement in cognition over time in TBI patients. Furthermore, only a small percentage of respondents with neither PTSD nor TBI showed change on the cognitive domain over time.

### Comparison to previous studies

Previous studies on measurement properties of the EQ-5D-5L did not investigate the cognitive domain, and focussed mainly on the comparison of the EQ-5D-5L with the EQ-5D-3L in a specific population, such as stroke patients, patients with psoriasis, cancer patients, and patients with hepatitis B [9–12, 30–32]. The studies by Poór et al. [30], Kim et al. [31] and Scalone et al. [32] found that ceiling effects decreased in the EQ-5D-5L compared to the EQ-5D-3L. In our study, we found high ceiling effects, especially 12 months post-trauma, most likely due to the fact that the majority of our study sample sustained mild to moderate trauma and most patients had recovered fully over 12 months. A previous study by Geraerds et al. [17] compared ceiling effects of the EQ-5D-3L and EQ-5D-3L+C in a sample of trauma patients in the Brabant region of the Netherlands who were admitted to the hospital due to injury. Their results are in agreement with our findings on the ceiling effects of the EQ-5D-5L and EQ-5D-5L+C, namely that fewer respondents reported perfect health when the EQ-5D+C is used. Furthermore, both the studies by Geraerds et al. [17] and Ophuis et al. [18] concluded that adding a cognitive domain slightly increased the explanatory power of the EQ-5D-3L. This is in line with the findings in this study, for the EQ-5D-5L. Another study that looked into the effect of adding a domain to the EQ-5D is the study by Swinburn et al. [33], who analysed the effect of adding an extra domain to the EQ-5D-5L for psoriasis patients. The results of their study showed that additional variance was captured by adding two extra domains for this specific population. This indicates that measurement of HRQL in specific patient populations might benefit from adding bolt-ons to the EQ-5D-3L as well as the EQ-5D-5L.

To our knowledge, responsiveness to change of the EQ-5D-5L+C has not been studied before. One study that analysed the responsiveness of the EQ-5D-3L in patients that underwent surgery for a hernia repair, varicose vein surgery, or cataract surgery determined the Paretian classification as well [26]. Results showed that in the group of patients who underwent hernia repair and the group of patients that underwent varicose vein surgery, health status of the majority improved. Relating these results to our own study is complicated, as different variants of the EQ-5D were used and study population differed. However, the explorative results of our study showed the same change over time: for the majority of the research population health status improved over time (subgroup of respondents with PTSD excepted, where more mixed change was found).

Even though it is difficult to compare the findings of our study with previous findings, we believe that our findings add information to the discussion on whether or not the five domains of the EQ-5D-5L are sufficient to measure HRQL. Especially in patients with diagnoses that are associated with cognitive impairments, such as TBI patients and patients with PTSD, the EQ-5D-5L+C outperformed the EQ-5D-5L; even though improvements were rather small. In addition, the findings of our study showed that in a specific population, replacing one of the EQ-5D domains with a bolt-on domain may increase the explanatory power. This could be due to domain dependency, which could be studied further in future research.

### Strengths and limitations

This study had strengths and limitations that need to be taken into account when interpreting the results. One of the strengths of our study was to have access to a large, longitudinal dataset of trauma patients. A second strength was that information on the presence of TBI and PTSD was available. Finally, the multi-criteria psychometric strategy that was adopted proved to be a useful approach to assess the benefit of adding the cognitive domain to EQ-5D.

An important limitation of our study was that the severity level of trauma appears to be low in the study population. Due to the absence of information on severity level of trauma of the respondents this suspicion cannot be supported with data. However, results showed that, compared to previous research, large ceiling effects were found, which could indicate that the severity of trauma of our research population was rather mild. Therefore, the study population might not be representative for a general trauma population.

Another limitation of our study was that the number of respondents with PTSD was low (107 (5.9%)). This may be the result of sampling bias, meaning that respondents with PTSD had a lower probability of participating in the study. A study by Geraerds et al. [34] studied a similar population of trauma patients in the Netherlands and found that responders were more severely injured than non-responders. In this study, we found that there were no respondents with PTSD that reported no problems, and that the percentage of respondents with positive change was highest for the PTSD group, compared to the TBI group and the group with neither TBI nor PTSD. This could indicate that only respondents with PTSD who suffered severe injuries participated in our study. Therefore, results with respect to PTSD should be interpreted with caution, as the PTSD population might not be representative. Possible outliers in the PTSD group will have had a large effect on the outcomes, and could have affected the results.

## Conclusion

In conclusion, adding a cognitive domain to the EQ-5D-5L decreased ceiling effects and improved the classification efficiency and the responsiveness of the EQ-5D-5L. Even though improvements in measurement properties were small, the results of a responsiveness analysis indicate that a cognitive domain is relevant, especially in respondents with TBI and/or PTSD, as a higher percentage of these respondents showed change on the cognitive domain. Cognition of respondents with TBI and/or PTSD changed between 6 and 12 months post-trauma for a larger percentage of the group than for the group of respondents with neither TBI nor PTSD. Since TBI and PTSD are both associated with cognitive problems, this underlines the relevance of adding the cognition bolt-on to the EQ-5D-5L when measuring HRQL in a population with cognitive problems.
